# Body cooling effects of immersion of the forearms in high-concentration artificial carbonic acid water at 25°C

**DOI:** 10.1186/s40101-020-0212-3

**Published:** 2020-02-04

**Authors:** Yuuki Tanaka, Hisaho Nagano, Akihiro Taimura

**Affiliations:** Graduate School of Fisheries and Environmental Sciences, Nagasaki University, 1-14 Bunkyo-machi, Nagasaki, 852-8521 Japan

**Keywords:** Heat stroke, Cooling, Artificial carbonic acid water, Exercise, Core temperature, Skin blood flow, Thermal sensation

## Abstract

**Background:**

This study examined the effects of immersion in stirred, high-concentration, artificial carbonic acid water on body cooling.

**Methods:**

Seven healthy male students (23 ± 2 years old) participated in the experiment. Signed informed consent was obtained from all subjects before the experiment. The subjects changed into shorts and T-shirts and entered an experimental room (with room temperature controlled at 30 °C and relative humidity maintained at 70%) at least 30 min before starting the experiment. After starting the experiment, the subjects were asked to rest on an exercise bike for 5 min and then pedal for 20 min. The exercise load was set to reach 50% of each subject’s presumed maximum oxygen intake at 5 min after starting exercise. Subjects then continued pedaling for 1 min to cool down. After this exercise, subjects sat on a chair and immersed forearms in tap water or artificial carbonic acid water (CO_2_ water) at 25 °C for 20 min. During immersion, tap water or CO_2_ water was stirred slowly with a pump. After immersion, subjects rested for 10 min. Skin temperature and skin blood flow (left forearm), as well as heart rate and ear canal temperature, were measured continuously. Thermal sensation and thermal comfort were measured intermittently.

**Results:**

Skin blood flow of the immersed forearms was higher in CO_2_ water than in tap water during immersion. The blood flow in the last 5 min (average at rest was 100%) was significantly higher in CO_2_ water (290.85 ± 84.81%) than in tap water (104.80 ± 21.99%). Thermal sensation and thermal comfort were not different between conditions. Ear canal temperature significantly declined more in CO_2_ water (− 0.56 ± 0.31 °C) than in tap water (− 0.48 ± 0.30 °C) during immersion.

**Conclusions:**

Our study suggests that immersion of the forearms in slowly stirred CO_2_ water at 25 °C reduces core temperature elevated by heat stress or exercise more effectively than does tap water at the same temperature. Immersion of the forearms in stirred CO_2_ water at 25 °C could be useful as a preventive measure against heat stroke from summer work or exercise.

## Background

Exertional heat stroke is a medical condition that occurs when the core temperature exceeds 40°C [1, 2]. High temperature and humidity result in inefficient heat radiation and suffering from hyperthermia during exercise easily occurs. Early recognition of exertional heatstroke and rapid on-site cooling of individuals with exertional heat stroke to a near-normal resting core temperature is important for survival and to minimize heat-related injury [1–3]. There are various methods of body cooling, including water immersion [2, 4–7], cooling by a fan [8, 9], applying ice packs [9], and ice slurry ingestion [10, 11].

Cold or ice water immersion is the most effective method for reducing core temperature in hyperthermic individuals, whereas treatment recommendations clearly suggest the implementation of cooling methods before the transportation of affected individuals [12]. Cold water immersion is an effective method with which to extract heat. However, some researchers have urged caution concerning the implementation of cold water immersion in hyperthermic people [4].

Casa et al. presented a rebuttal to some arguments raised against this treatment for exertional heat stroke [2]. One emphasis was to contest the possibility that cold-induced thermogenesis promotes heat production and storage, thereby delaying cooling. Shivering will occur, with reduced sensitivity in preheated people [13]. Cold water immersion should be avoided for hyperthermic individuals because this is not only unpleasant, but it may result in cardiovascular failure (cold shock) in some high-risk individuals [4]. Cooling around the active muscle is considered to be a site that can control the rise in body temperature during intense exercise, as well as cooling the entire body, and can effectively suppress the rise in body temperature during exercise. However, excessive cooling around the active muscle may cause the temperature of the active muscle to decrease, which may have a negative effect on exercise performance [14]. Water immersion at 26 °C results in similar overall core cooling rates to those measured in much cooler temperature conditions [5].

Recent data have indicated that immersing the lower legs in stirred 25°C/30°C carbonic acid water (CO_2_ water) can suppress vascular constriction of the skin and reduce the core temperature raised by heat stress/exercise more effectively than stirred tap water of the same water temperature [6]. These results suggest that CO_2_ water immersion is effective in reducing core temperature by exercise while maintaining skin blood flow. However, supplying a large volume of water in all occupational settings is not always possible or practical [8].

Therefore, this study aimed to examine the effects of immersion of the forearms in a relatively small amount of cold (25 °C), stirred, high-concentration artificial CO_2_ water on body cooling. We hypothesized that these conditions suppress skin vascular constriction and cold stimulation, thus reducing the core temperature while maintaining skin blood flow.

## Methods

### Participants

Seven healthy male students (mean age 23 ± 2 years, mean height 1.71 ± 0.04 m, mean body weight 64.3 ± 5.1 kg, and mean body mass index 22.1 ± 1.7 kg/m^2^) participated in this study. Our experiments were approved by the Ethics Committee of the Faculty of Environment Science, Nagasaki University. All participants signed an informed consent form before our experiments began.

### Preliminary measurements

To determine maximum oxygen intake (VO_2_max), each participant performed a progressive exercise test on a cycle ergometer (AEROBIKE 75XL; Combi Co., Ltd., Tokyo, Japan) at 26 °C and 50% relative humidity on their first visit to the laboratory. The protocol consisted of a progressive, gradual increase in exercise load until attaining 75% of the maximum heart rate. The participants were asked to maintain a pedal cadence of 50 revolutions per minute throughout the progressive exercise test.

### Experimental procedure

In a randomized, crossover design, all of the participants performed two trials each as follows. Participants had immersion in tap water or a high concentration of artificial CO_2_ water after exercise. The participants were instructed to avoid strenuous exercise and consumption of alcohol from at least 24 h before the experiment. Each participant arrived at the laboratory after having refrained from eating and drinking any type of beverage for 2 h. For each participant, the two trials were commenced at the same time to control for circadian variation in core temperature and were separated by 2–5 days.

**Fig. 1:**
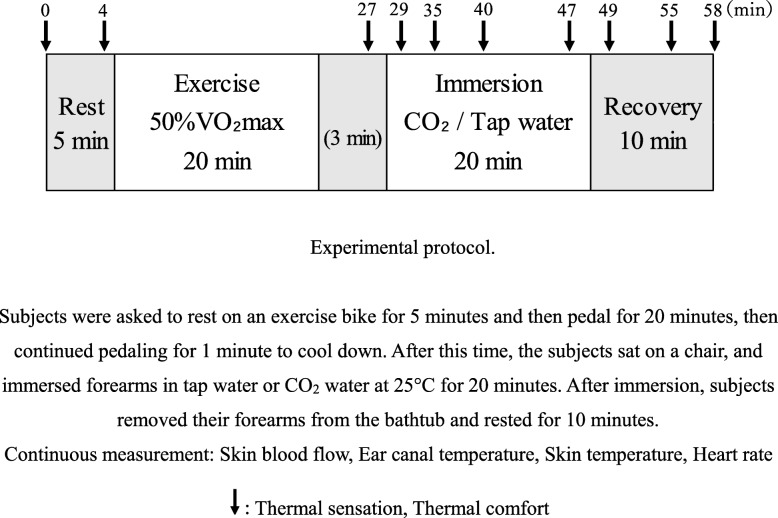
Experimental protocol. Subjects were asked to rest on an exercise bike for 5 min and then pedal for 20 min, then continued pedaling for 1 min to cool down. After this time, the subjects sat on a chair and immersed forearms in tap water or CO_2_ water at 25 °C for 20 min. After immersion, subjects removed their forearms from the bathtub and rested for 10 min. Continuous measurement: skin blood flow, ear canal temperature, skin temperature, heart rate. Arrow down: thermal sensation, thermal comfort

Upon arrival at the laboratory, the participants changed into shorts and T-shirts and had body weight measured before they entered a climate-controlled room (30 °C and 70% relative humidity). After entering the experimental room, at least 30 min before the start of the experiment, participants were asked to rest on an exercise bike for 5 min and then pedal for 20 min. The exercise load was set to reach 50% of each subject’s presumed VO_2_max at 5 min after starting the exercise. Subjects then continued pedaling for 1 min to cool down. After this time, the subjects sat on a chair and immersed forearms in tap water or CO_2_ water at 25 °C for 20 min. The experimental protocol is shown in Fig. 1. During immersion, tap water or CO_2_ water was stirred by a stirrer pump that was attached to a thermostatic bathtub (TR2; Iuchiseieido Co., Ltd., Osaka, Japan) with 20 L of water. After 20 min of immersion, subjects removed their forearms from the bathtub and rested for 10 min. CO_2_ water was made by an artificial carbonic water manufacturing device (CARBO THERA mini; Mitsubishi Rayon Engineering Co., Ltd., Tokyo, Japan). The CO_2_ water contained a high concentration of CO_2_ (> 1000 ppm). The CO_2_ concentration in the water was measured using a pH meter (pHScan WP3; AS ONE Co., Ltd., Osaka, Japan) and by converting the pH of the water into CO_2_ concentration.

### Measurements

For physiological indices, ear canal temperature, skin temperature, skin blood flow, heart rate, sweat rate, thermal sensation, and thermal comfort were measured. Core temperature was measured at an ear canal using a thermistor sensor (LT-2N-13; Gram Co., Ltd., Saitama, Japan). Skin temperature was measured at the left forearm using thermistors (RXK67; TECHNO SEVEN Co., Ltd., Tokyo, Japan). Skin blood flow was measured at the left forearm using a laser Doppler flowmeter (ALF21D; Advance Co., Ltd., Tokyo, Japan). All thermistors and flowmeters were connected to a data collection device (LT-8A or LT-200SB; Gram Co., Ltd., Saitama, Japan) and recorded every 5 s. Heart rate was monitored using a heart rate monitor (ACCUREX Plus; Polar Co., Ltd., Kempele, Finland). Sweat rate was estimated using the following formula: body weight before the experiment − body weight after the experiment. Body weight was measured using a weighing machine (ID 1 Plus; Mettler Toledo Co., Ltd., Tokyo, Japan). Subjective thermal sensation and comfort were measured at 0, 4, 27, 29, 35, 40, 47, 49, 55, and 58 min after the experiment started using a scale (0 = very cold to 8 = very hot) (1 = comfort to 5 = uncomfortable). Ear canal temperature, skin temperature (forearm), and skin blood flow (forearm), as well as heart rate were measured continuously, and thermal sensation and thermal comfort were measured intermittently. Water temperature was measured in the water in the bathtub, which was 5.5 cm in depth, using thermistors (RXK67; TECHNO SEVEN Co., Ltd. Tokyo, Japan).

### Data analysis

Results are shown as mean ± standard deviation. Statistical comparisons of results for ear canal temperature, skin temperature, skin blood flow, and heart rate were made using two-factor (condition and time) ANOVA with repeated measures, followed by the signed rank-sum test. Sweat rate, thermal sensation, thermal comfort, and water temperature were compared between conditions by using the signed rank-sum test. Statistical significance was accepted at *P* < 0.05. Statistical analysis was performed using Excel StatcelQC software (version 2016 for Windows).

## Results

### Skin blood flow

**Fig. 2:**
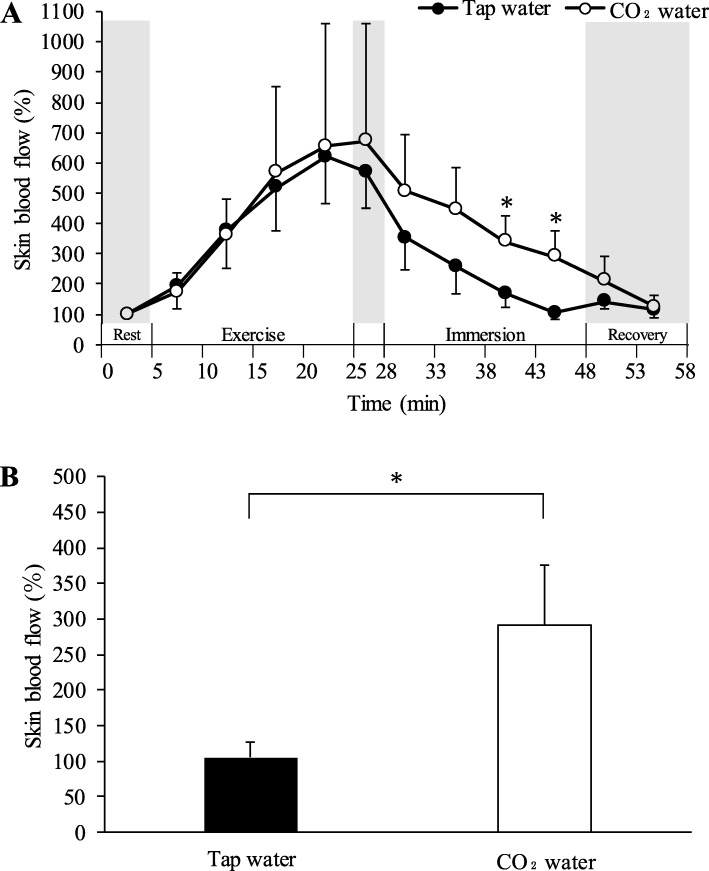
Time-course of skin blood flow of the left forearm during the experiment (**a**). Skin blood flow relative to the resting value for the last 5 min during immersion (**b**). All values are expressed as mean ± standard deviation. *P < 0.05 between tap water and CO_2_ water

Skin blood flow relative to the resting (before exercise) value for the last 5 min during immersion of the forearms is shown in Fig. 2. Skin blood flow of the forearm was higher in CO_2_ water than in tap water during immersion. The skin blood flow in the last 5 minutes (average at rest was 100%) was significantly higher in CO_2_ water (290.85 ± 84.81%) than in tap water (104.80 ± 21.99%) (*P* < 0.05).

### Ear canal temperature

**Fig. 3:**
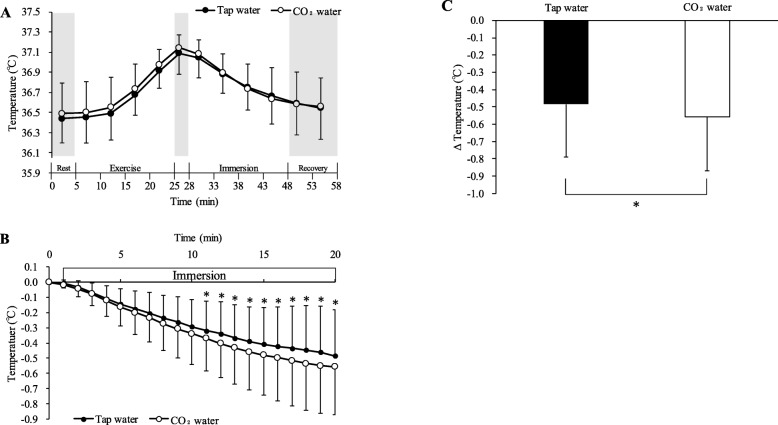
Time-course of ear canal temperature during the experiment (**a**). Changes in ear canal temperature from before immersion (**b**). Changes in ear canal temperature before and after immersion (**c**). All values are expressed as mean ± standard deviation. *P < 0.05 between tap water and CO_2_ water

Ear canal temperature during rest before exercise was not significantly different between conditions (CO_2_ water, 36.49 ± 0.31°C; tap water, 36.44 ± 0.24 °C). Ear canal temperature gradually rose throughout exercise, and the ear canal temperature in the last 5 min during exercise was 36.98 ± 0.15 °C in CO_2_ water and 36.91 ± 0.17 °C in tap water. There was no significant difference in ear canal temperature between conditions. The change in ear canal temperature before and after immersion is shown in Fig. 3. Ear canal temperature declined more in CO_2_ water (− 0.56 ± 0.31 °C) than in tap water (− 0.48 ± 0.30 °C) during immersion. Ear canal temperature in CO_2_ water was significantly reduced from baseline to 20 min compared with tap water (*P* < 0.05).

### Skin temperature

Skin temperature during rest before exercise was not significantly different between conditions (CO_2_ water, 33.94 ± 0.40 °C; tap water, 33.64 ± 0.57 °C). Skin temperature gradually rose throughout exercise, and skin temperature of the forearm in the last 5 min during exercise was 35.87 ± 0.58 °C in CO_2_ water and 35.58 ± 1.04 °C in tap water. There was no significant difference in skin temperature between conditions. Skin temperature of the forearm in the last 5 min during immersion tended to be higher in CO_2_ water (27.23 ± 0.36 °C) than in tap water (27.04 ± 0.37 °C) (*P* = 0.09).

### Sweat rate

The change in body weight before and after the experiment was not significantly different between conditions , but the sweat rate tended to be lower in CO_2_ water (0.24 ± 0.06 kg) than in tap water (0.28 ± 0.09 kg) during the experiment (*P* = 0.09).

### Heart rate

**Fig. 4:**
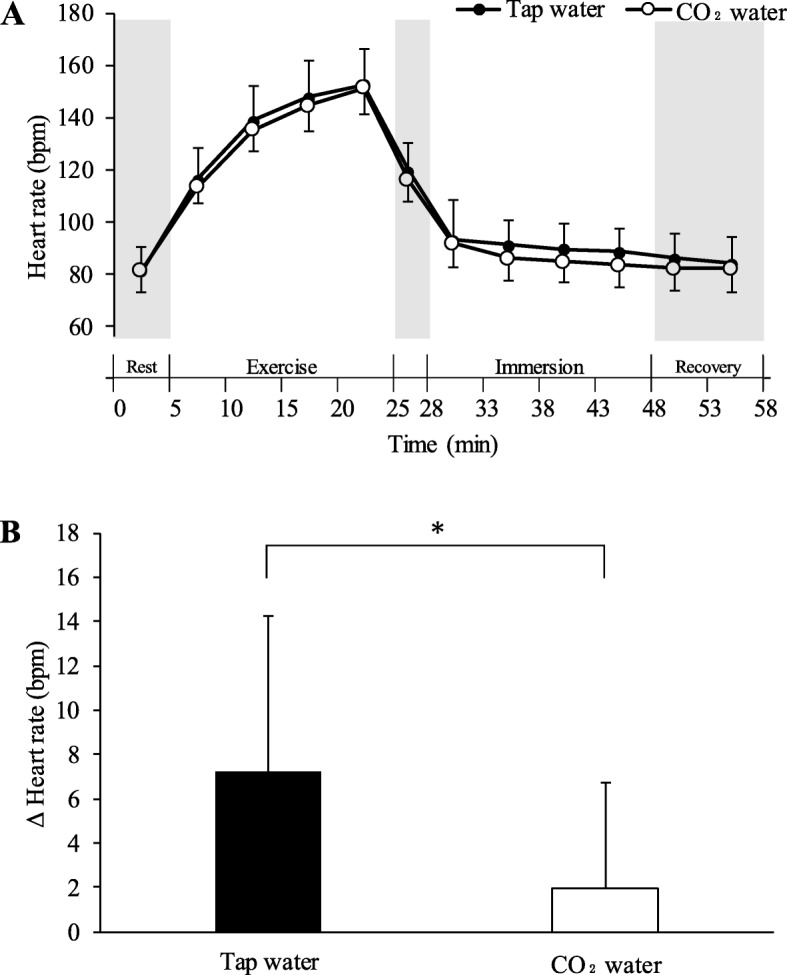
Time-course of heart rate during the experiment (**a**). Changes in heart rate from rest to the last 5 min during immersion (**b**). All values are expressed as mean ± standard deviation. *P < 0.05 between tap water and CO_2_ water

Heart rate during rest before exercise was not significantly different between conditions (CO_2_ water, 81.43 ± 8.75 bpm; tap water, 81.23 ± 9.39 bpm). Heart rate gradually rose throughout exercise, and in the last 5 min during exercise, it was 151.17 ± 10.24 bpm in CO_2_ water and 152.69 ± 13.34 bpm in tap water. There was no significant difference in heart rate between conditions. The heart rate in the last 5 min during immersion was 83.38 ± 8.57 bpm in CO_2_ water and 88.43 ± 9.21 in tap water, and the change from rest before exercise was significantly lower in CO_2_ water (1.96 ± 4.75 bpm) than in tap water (7.2 ± 7.06 bpm) (Fig. 4).

### Thermal sensation and comfort

Thermal sensation during rest before exercise was not significantly different between conditions (CO_2_ water, 4.00 ± 0.00; tap water, 4.13 ± 0.33). Thermal sensation gradually rose throughout immersion, but at the end of immersion, there was no difference between conditions (CO_2_ water, 3.00 ± 0.96; tap water, 3.00 ± 0.65). Thermal comfort during rest before exercise was not significantly different between conditions (CO_2_ water, 3.03 ± 0.08; tap water, 3.00 ± 0.00). Thermal comfort remained stable throughout immersion, but at the end of immersion, there was no difference between conditions (CO_2_ water, 2.36 ± 0.64; tap water, 2.57 ± 0.73).

### Water temperature

Water temperature in the bathtub before immersion was not significantly different between conditions (CO_2_ water, 25.01 ± 0.04 °C; tap water, 25.01 ± 0.03 °C). Water temperature gradually rose throughout immersion (CO_2_ water, 26.82 ± 0.24 °C; tap water, 26.74 ± 0.26 °C). There was no significant difference in water temperature between conditions, but water temperature tended to be slightly increased more with CO_2_ water (1.81 ± 0.25 °C) than in tap water (1.73 ± 0.25 °C) (*P* = 0.09).

## Discussion

In the current study, we found that skin blood flow was significantly higher in CO_2_ water than in tap water. CO_2_ water suppressed the decrease in blood flow compared with tap water. This finding indicated that CO_2_ water at 25 °C attenuated skin vasoconstriction in the forearm. Even in conditions where the forearm was immersed in CO_2_ water at 25 °C, contraction of skin blood vessels was able to be suppressed.

In our study, the ear canal temperature was decreased significantly more during immersion in stirred CO_2_ water than in tap water at 25 °C. This result suggests that immersing the forearm in stirred CO_2_ water at 25 °C suppresses vascular constriction of the skin and reduces core temperature elevated by heat stress exercise more effectively than stirred tap water of the same water temperature. Therefore, the body can be cooled in a simple manner by using this method. Our finding of a significant reduction in core temperature is important. If the core temperature rises at the end of exercise or work, CO_2_ water immersion has a greater cooling effect than does tap water. The forearms correspond to 10% of the body surface area. If the immersion area is increased, the core temperature decreases. The purpose of this study was to investigate a relatively easy way and practical method of cooling the body. Therefore, we examined immersion of the forearms, which only requires a relatively small amount of water.

When the lower legs are immersed, the tympanic temperature is significantly decreased more during immersion in stirred 25 °C CO_2_ water (− 0.70 ± 0.25 °C) than in tap water (− 0.58 ± 0.29 °C) [6]. The lower legs correspond to 20% of the body surface area. In our study, ear canal temperature declined more in CO_2_ water than in tap water during immersion. This finding is difficult to compare with previous data because of different measurement methods and subjects. However, a difference in immersion area affects the extent of a decrease in core temperature. The core temperature might decrease as the immersion area increases.

A high skin temperature under CO_2_ water conditions indicates that the temperature gradient between the skin and water can be maintained. In our study, during immersion, there were no differences in thermal sensation and thermal comfort between conditions. When the lower legs are immersed in CO_2_ water at 30 °C without stirring, CO_2_ water is significantly warmer than tap water in thermal sensation, and thus a cooling sensation-suppressing effect is obtained [6]. Stirred water can maintain a temperature gradient and immersed parts always feel a temperature difference. Additionally, forearms have a higher density of cold spots than do the lower legs [15]. Therefore, the cold sensation-suppressing effect of CO_2_ water is less effective by inhibition of more cold receptors in the forearm. Additionally, heat dissipation is thought to be achieved by vasodilation of the skin as a result of the sweat rate volume becoming lower in the CO_2_ condition. Because sweating deprives the body of water, this method provides less burden on the body in terms of physiological fluid retention. Suppression of a rise in core temperature affects the degree of skin blood flow more than perspiration during exercise at a temperature of 31 °C [16]. Immersion in CO_2_ water is efficient because it can promote heat dissipation by vasodilation (suppressing elevation), which has a greater effect than sweating.

Heart rate during immersion was lower in CO_2_ water than in tap water. Hashimoto and Yamamoto showed that vascular resistance of the immersed skin of rats in CO_2_ water was significantly less than that in tap water [17]. Therefore, vascular conductance was increased. In their study, with increased skin blood flow, there was a decrease in pressure in the cardiovascular system with a decrease in heart rate. Therefore, blood pressure might be reduced during immersion and this might increase vascular conductance. We aimed to examine the effects of immersion in cold, stirred, CO_2_ water at a high concentration on body cooling. After exercising with a bicycle ergometer, we found that CO_2_ water significantly reduced the core temperature compared with tap water. This finding suggests that, by maintaining a high skin blood flow by stirred 25 °C CO_2_ water, a lot of heat is present on the skin’s surface and this generates stirred flow to maintain a gradient between skin temperature of the immersed body part and water temperature. The above-mentioned finding occurred by increasing the amount of heat dissipation and decreasing the core temperature. Therefore, our findings suggest that body cooling can be efficiently performed by increasing the skin blood flow rate and increasing the amount of heat dissipation by using CO_2_ water. The net rate of heat loss from the surface of the skin to the surrounding environment is the sum of heat exchange via conduction, convection, radiation, and evaporation [18].

In an emergency situation, the patient needs to be immediately immersed in the most readily available cool water and then cooler water must be sought [4]. This study focused on prevention among heat stroke measures. There are various methods of body cooling, and some previous studies are related to preventing heat stroke [6, 8–10]. Elevation in the rate of production of metabolic heat while working, combined with high ambient temperature and humidity, can lead to progressive increases in body heat content. Prolonged working may lead to heat illness and eventually death. It is necessary to lower the core temperature even a little to avoid heat stress. Furthermore, attainment of critical core temperature has been suggested to be one of the main limiting factors inhibiting endurance exercise performance [19, 20]. Naito et al. reported that rectal temperature in the ice ingestion and long rest interval trial continued to drop until 20 min after ingestion and resulted in greater reduction in rectal temperature (− 0.55 ± 0.07 °C) than in trials on the short rest interval (− 0.36 ± 0.16 °C) and cold water ingestion (− 0.11 ± 0.14 °C). The cycling time to exhaustion was significantly different under the three conditions. This results indicate that precooling by ice ingestion and long rest interval reduced rectal temperature and heat storage before exercise, thereby enhancing exercise capacity in hot environments as compared with cold water ingestion [21]. Their study focused on precooling for increase heat storage before exercise, whereas this study aimed to examine the effects on post-cooling by immersion in CO_2_ water after exercise. Mawhinney et al. reported that whole-limb immersion in 8 °C and 22 °C water after exercise decreased rectal temperature. Rectal temperature was reduced over time and to the greatest extent 30 min after immersion (8°C, − 0.7 ± 0.3 °C; 22 °C, − 0.6 ± 0.2 °C) [22]. The extent of tissue damage and physiological malfunctions depend not only on the degree of hyperthermia but also on the duration in which the body remains at high temperature [23]. The main objective in the treatment of hyperthermia is therefore to reduce the body temperature to a safe level. In this study, ear canal temperature was significantly reduced in CO_2_ water immersion compared with tap water immersion at the same temperature and duration. This result suggested that CO_2_ water reduces body hyperthermia more efficiently than tap water, demonstrating its applicability as a heatstroke measure. For a cooling method that is more practical and convenient, future investigation should examine the effective duration of the immersion, as well as the water temperature, immersed body part, and effect of CO_2_ water concentration on the inhibition of heat stress, for application in a variety of occupational and industrial settings.

## Conclusions

Our study shows that immersion of the forearms in high-concentration artificial CO_2_ water causes a greater decline in core temperature than does tap water. Our findings suggest that a high concentration of artificial CO_2_ water at a temperature lower than skin temperature suppresses vasoconstriction of the skin of the immersed body part by maintaining the skin blood flow rate higher than that with the same temperature of tap water. CO_2_ water efficiently decreases core temperature. Therefore, immersion of the forearms in stirred, 25 °C, high-concentration, artificial CO_2_ water might result in cooling efficiency without the burden of cold water stimulation. This method will be useful as a preventive measure against heat stroke from summer work or exercise.

## Data Availability

The datasets used and/or analyzed during the current study are available from the corresponding author on reasonable request.

## Abbreviations

bpm: Beats per minute
CO_2_ water: Carbonic acid water
VO_2_max: Maximum oxygen intake
